# Nematic Alignment of Composite Silver-Coated Gold Nanorods and Cellulose Nanocrystals

**DOI:** 10.3390/nano15201594

**Published:** 2025-10-19

**Authors:** Chenxi Li, Julian Evans, Bo Gao, Guancheng Shen, Sailing He, Weixing Yu

**Affiliations:** 1 Key Laboratory of Spectral Imaging Technology, Xi’an Institute of Optics and Precision Mechanics, Chinese Academy of Sciences, Xi’an 710119, China; lichenxi@opt.ac.cn (C.L.); gaobo_101@opt.ac.cn (B.G.); 2 Center for Optical and Electromagnetic Research, State Key Laboratory for Modern Optical Instrumentation, Zhejiang University, Hangzhou 310058, China; julian.evans@colorado.edu; 3 Xi’an Modern Chemistry Research Institute, Xi’an 710065, China; gc_shen@outlook.com; 4 Ningbo Research Institute, Zhejiang University, Ningbo 315100, China; 5 Department of Electromagnetic Engineering, School of Electrical Engineering, Royal Institute of Technology (KTH), S-100 44 Stockholm, Sweden; 6 Center of Materials Science and Optoelectronics Engineering, University of Chinese Academy of Sciences, Beijing 100049, China

**Keywords:** cellulose nanocrystals, silver-coated gold nanorods, self-assembly, nematics

## Abstract

Cellulose nanocrystals (CNCs) have been extensively studied for their ability to maintain liquid crystal (LC) order within solid films, providing a robust template for the self-assembly of plasmonic nanorods (NRs) and the construction of nanostructures. The self-assembly mechanism of NRs combined with uniaxially nematic CNC LCs has long attracted considerable attention. In this study, we investigated the influence of pH and aspect ratio on the self-assembly of composite NR–uniaxial nematic CNC systems. The phase diagram indicates that the uniaxial nematic phase of CNCs becomes more stable at higher pH, while it is more sensitive to disturbance from NRs with smaller aspect ratios. Furthermore, a composite effective excluded volume model was developed, in which the interaction between NRs and CNCs is incorporated, and the effective excluded volume is governed by both the effective CNC diameter and the NR aspect ratio. This study elucidates the influence mechanism of pH and aspect ratio on the self-assembly of composite NR–uniaxial nematic CNC systems, in good agreement with experimental observations. Our results provide fundamental insights into the utilization of uniaxial nematic CNC LCs as templates for fabricating novel nanomaterials and nanostructures, and deepen understanding of the mechanisms governing such composites.

## 1. Introduction

Composite plasmonic nanorod (NR)–liquid crystal (LC) systems integrate tunable surface plasmonic properties with the optical birefringence of LCs, exhibiting great potential for advanced photonic nanomaterials and multifunctional nanostructures [1,2,3]. Composite plasmonic NR–thermotropic LCs are prone to sedimentation and show structural instability as thermotropic LCs can only maintain their orientation in the fluid state [4,5]. Cellulose nanocrystal (CNC) LCs can retain their orientation in solid films after drying, serving as robust templates for the self-assembly of plasmonic NRs [6,7]. During the drying process, CNC LCs transition into a glassy state, effectively freezing the long-range LC order and permanently locking this anisotropic structure within the solid film [8,9]. CNCs, which are rod-like nanoparticles obtained through the acid hydrolysis of natural cellulose, possess negatively charged surfaces and can form LC phases above a critical concentration [10,11]. CNCs can generally be classified into two types based on their origin: plant-derived (e.g., from wood or cotton) and bacterial [12]. Plant-based CNCs typically exhibit similar phase behaviors because of their comparable diameters and lengths, whereas bacterial CNCs are usually longer and display distinct structural characteristics [13]. CNC LCs typically exhibit chiral nematic ordering but can also form uniaxial nematic phases, which generally require additional conditions such as larger CNC aspect ratios and precise pH control [14,15]. The self-assembly and phase behavior of composite NR–chiral nematic CNC LC systems have been extensively studied [16,17]. Composite NR–chiral nematic CNCs are commonly used as Bragg reflection structure [18,19], while their uniaxially aligned structures show lower anisotropy due to the intrinsic helical orientation of CNCs [20]. Composite NR–uniaxial nematic CNC LCs with long-range parallel orientation show great potential for fabricating anisotropic plasmonic nanostructures such as polarizers, color filters, and so on [21,22]. However, the phase behavior of uniaxial nematic composites remains far less explored.

Understanding the underlying physical mechanisms of composite NR–uniaxial nematic CNC systems remains a major challenge, which limits their further development and practical optical applications. Liu et al. investigated the phase behavior of gold NRs co-dispersed in uniaxial nematic CNC liquid crystals (LCs), under the assumption that the CNC alignment remained unaffected by the gold NRs [23]. Peranza et al. developed a bidisperse Onsager model predicting that NRs with smaller aspect ratios exert stronger disruptive effects on LC alignment [24]. However, these analyses primarily focused on individual NRs and did not systematically explore the critical influence of NR aspect ratio on composite phase behavior. Campbell et al. incorporated gold NRs into uniaxial nematic CNC LCs and aligned the composite system via shear force, demonstrating its feasibility as a plasmonic polarizer operating around 680 nm [25]. Hess et al. employed 3D printing to orient a composite of gold NRs and CNCs, achieving spatially programmable, polarization-dependent plasmonic properties near 650 nm [26]. In addition, Li et al. doped silver nanowires (AgNWs) into uniaxial nematic CNC LCs and aligned the composite through shearing, exhibiting polarization-dependent optical properties at 438 nm [27]. Zhang et al. fabricated tunable structural color elements using hybrid GNR/CNC films, in which color tuning was achieved through layer-by-layer rotational stacking [28] and uniaxial stretching [29]. These reported NR–CNC composite systems operate at a single wavelength, or their operational wavelength is tuned only through external fields, limiting their integration level and tunability. Therefore, bridging this knowledge gap regarding the influence of NR aspect ratio on composite NR–uniaxial nematic CNC LCs is crucial for elucidating the phase mechanisms of such composites and provides a strong foundation for developing highly tunable and integrated plasmonic nanostructures.

In this study, we investigated the influence of pH and aspect ratio on the self-assembly of composite NR–uniaxial nematic CNC systems. Twelve composite NR–CNC LCs were systematically prepared, in which both the pH-dependent uniaxial nematic ordering of CNCs and the aspect ratio of NRs were varied. Polarized optical microscopy (POM) was employed to characterize the CNC LC domains and textures. The phase diagram indicates that the uniaxial nematic phase of CNCs becomes more stable at higher pH, while it is more sensitive to disturbance from NRs with smaller aspect ratios. Furthermore, a composite effective excluded-volume model was developed, in which the interactions between NRs and CNCs are incorporated, and the effective excluded volume is governed by both the effective CNC diameter and the NR aspect ratio. This study elucidates the influence mechanism of pH and aspect ratio on the self-assembly of composite NR–uniaxial nematic CNC systems, in good agreement with experimental observations.

## 2. Materials and Methods

### 2.1. Materials

**Figure 1:**
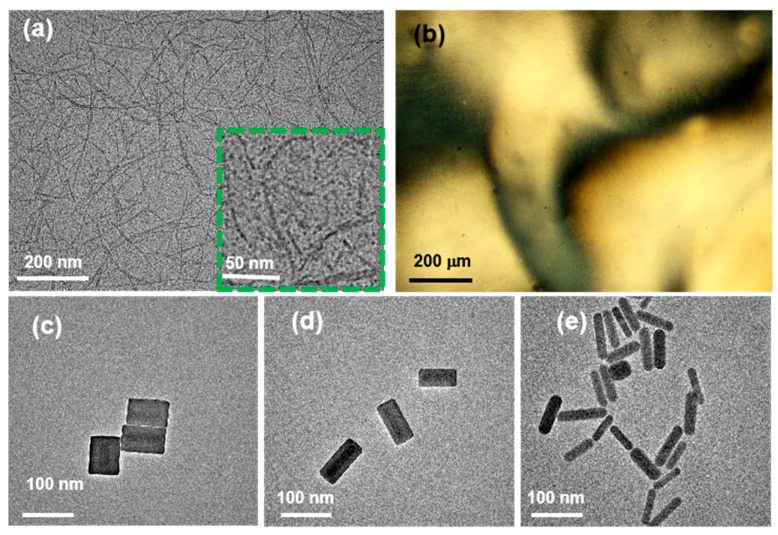
(**a**) TEM image of CNCs, where the green box indicates the enlarged TEM image of CNCs (**b**) POM image of the uniaxial nematic CNC LCs. (**c**–**e**) TEM images of three types of NRs with aspect ratios of (**c**) 1.3, (**d**) 2, and (**e**) 3.4.

Figure 1a shows a TEM image of CNCs with a rod-like morphology, an average length of 200 nm, an average width of 10 nm, and an aspect ratio (*AR* = *L*/*D*) of 20. Figure 1b presents a POM image displaying schlieren texture, confirming the formation of a uniaxial nematic phase. Figure 1c–e show TEM images of three types of NRs, all exhibiting rod-like morphologies with similar average lengths of 80 nm. Their diameters are approximately 60, 40, and 20 nm, respectively (*D*′_1_ ≈ 60 nm, *D*′_2_ ≈ 40 nm, *D*′_3_ ≈ 20 nm), resulting in decreased aspect ratios (*AR*′_1_ ≈ 1.3 *AR*′_2_ ≈ 2, *AR*′_3_ ≈ 3.4).

These commercial NRs exhibit tunable plasmonic extinction peaks at 514 nm, 630 nm, and 750 nm, respectively, which are governed by their aspect ratios. Such tunability demonstrates their potential for applications in polarization-sensitive nanomaterials and nanostructures. The relatively large diameters of the NRs introduce more pronounced disturbances within the composite system, exerting a stronger influence on its phase behavior. Conversely, increasing the NR length (e.g., to several hundred nanometers) could also significantly affect the phase behavior, whereas shorter NRs impose only a minor effect [24]. Therefore, these three types of commercial NRs were selected for incorporation into uniaxial nematic CNC LCs to fabricate the composite systems.

### 2.2. Preparation of Composite NR–Uniaxial Nematic CNC Systems

**Figure 2:**
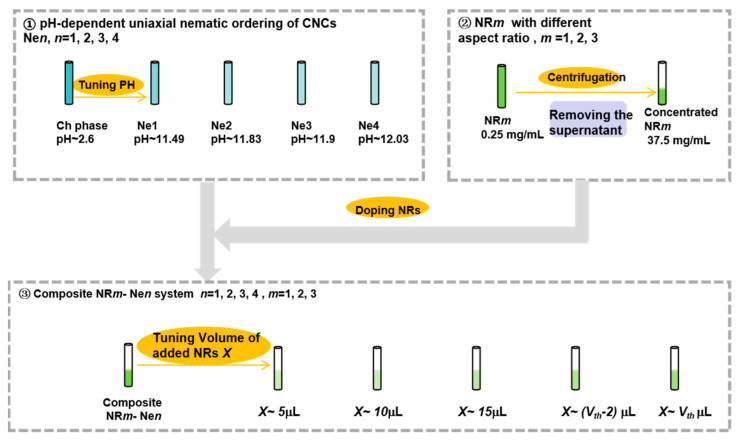
Schematic illustration of the preparation of composite NR–uniaxial nematic CNC systems with different pH values and NR aspect ratios.

Figure 2 illustrates the preparation process of composite NR–uniaxial nematic CNC systems with varying pH-dependent uniaxial nematic ordering of CNCs and different NR aspect ratios. As shown in Figure 2, the fabrication of these composites involves the following steps: First, uniaxial nematic CNC LCs were prepared using the pH control method based on our established method [14]. Specifically, 18 μL, 19.5 μL, 22 μL and 24 μL of 2 mol/L NaOH solutions were added into 1 mL of 5% chiral nematic CNC LCs (supplied by Nanjing Renewable Energy eco Materials Co. (Nanjing, China), zeta potential ≈ −30 mV), yielding four uniaxial nematic CNC LC samples designed as Ne1, Ne2, Ne3 and Ne4, with pH value of 11.49, 11.83, 11.9 and 12.03, respectively. Second, three sets of 1.5 mL NR suspensions (NR1: Au180514, NR2: Au180633, NR3: Au180750, supplied by Beijing Zhongkekeiming DaoJin Technology Co., Beijing, China) were centrifuged at 6000 rpm for 5 min. The pH of the NR suspension was measured to be approximately 7.0. At this near-neutral pH, the NRs were stabilized by a cetyltrimethylammonium bromide (CTAB), which imparted a positive surface charge (zeta potential ≈+35 mV). The supernatant was removed, and the NRs were concentrated to 37.5 mg/mL. Third, the concentrated NR suspensions were added to 100 μL of the uniaxial nematic CNC LCs. Various composite samples were prepared by adding different volumes of NRs (donated as *X*), followed by stirring for 2–3 min until a homogeneous mixture was obtained. Finally, twelve composite NR–uniaxial nematic CNC systems were prepared to study their self-assembly behavior (Table 1). These samples, fabricated using the above procedures, encompassed different pH-dependent uniaxial nematic CNC orderings (Ne1: 11.49, Ne2: 11.83, Ne3: 11.9, and Ne4: 12.03) and NR aspect ratios (NR1: 1.3, NR2: 2, and NR3: 3.4). In these composite systems, the volume of the uniaxial nematic CNC LCs was fixed as 100 μL, while the added volume of NRs (donated as *X*) gradually increased from 5 μL until the CNCs exhibited an isotropic phase.

### 2.3. Characterization

The morphology and dimensions of CNCs and NRs were characterized using a double spherical aberration-corrected transmission electron microscopy (FEI, Themis Z, Hillsboro, OR, USA). The pH of CNC dispersions was measured with a portable pH meter (Shanghai Sanxin Instrument Factory, PH60S-Z. Shanghai, China) equipped with a sharp-tip electrode, which had a resolution of 0.01 pH and an accuracy of ±0.01 pH units. The zeta potential of the samples was measured using a Zetasizer Nano ZS (Malvern, UK) based on the dynamic light scattering technique. POM images were obtained with a microscope (Novel optics, NMM-820TRF, Zhejiang, China) to examine the LC textures and phase behavior of the composite NR–uniaxial nematic CNC LC systems.

### 2.4. Numerical Simulation Approach Based on the Onsager Excluded-Volume Model

**Figure 3:**
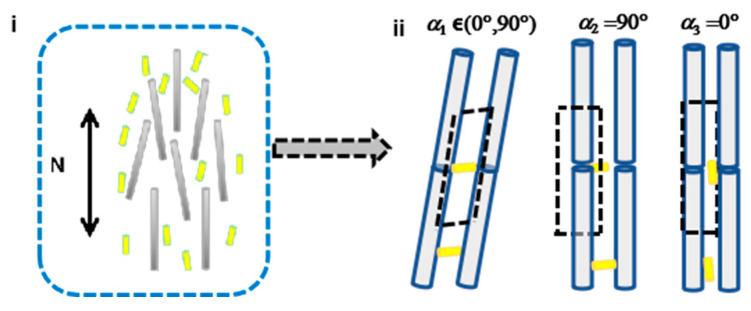
Schematic illustration of the excluded volume in the composite NR–uniaxial nematic CNC LC system. (**i**) Structure schematic of NRs embedded within uniaxial nematic ordered CNCs, where yellow and silver rods represent NRs and CNCs, and the black arrow indicates the director N of uniaxial nematic CNC ordering. (**ii**) Schematic of an NR–CNC unit. The black dashed quadrilateral outlines the effective excluded volume of the composite unit, with the angle α donating the relative orientation between CNC and NR. From left to right, the images illustrate the excluded volume at relative angles of $\alpha_{1}\in (0{}^{\circ},90{}^{\circ})$, $\alpha_{2}=90{}^{\circ}$ and $\alpha_{3}=0{}^{\circ}$.

Figure 3 presents a schematic illustration of the excluded volume in the composite NR–CNC system. Figure 3i shows the composite structure in which NRs are embedded within the uniaxially nematic CNC LC, while Figure 3ii depicts the corresponding unit schematic. In Figure 3i, the yellow and silver rods represent the added NRs and CNCs, respectively, and the black arrow indicates the director (**N**) of the uniaxial nematic ordering of CNCs. Figure 3ii illustrates that the black dashed quadrilateral denotes the excluded volume of the unit cell, and the angle between the CNC and NR orientations reflects the effect of their mutual alignment. Additionally, Figure 3ii shows NR–CNC unit schematics at different relative orientation angles.

For a given relative angle α∈(0°,90°), the excluded volume V of composite NR–CNC systems can be calculated based on the bidisperse Onsager model [23,30,31], and is given by:

(1) $$V=\left(D+D^{\prime }\right)L^{\prime }L\;cos$$

where *D* and *L* denote the diameter and length of CNCs, where $D^{\prime }$ and L′ denote the diameter and length of NRs, respectively. In addition, when the relative angle α is 90°, the excluded volume V reaches its maximum value, which can be calculated by

(2) $$V_{⊥}=(D+D\prime )\;L\prime L$$

Conversely, when the angle α approaches 0°, the excluded volume V is minimized and can be expressed by

(3) $$V_{∥}=\pi {\left(D+D\prime \right)}^{2}\;L/4$$

Considering the values in Equations (2) and (3), the excluded volume for parallel alignment is smaller than that for perpendicular alignment ($V_{∥}<V_{⊥}$). Therefore, the doped NRs in these composite CNC systems tend to align parallel to the CNC direction (**N**) to minimize the excluded volume. Consequently, Equation (3) governs the phase transition of CNCs from the uniaxial nematic phase to the isotropic phase for CNCs. Moreover, according to the Onsager threshold [30], nanorods require an aspect ratio greater than 4 (AR_th_ = 4) to form a LC phase.

To establish a general theoretical framework for understanding the phase behavior of plant-derived CNC composite systems, a series of simulations was performed. The simulation parameters *L*, $D^{\prime }$ and L′ were extracted from TEM images, and the effective diameter *D*_eff_ was used instead of *D* to account for the electrostatic interactions of CNCs. Since both our reported results [14] and the CNCs used in this study are plant-derived, they exhibit consistent phase behavior. The effective excluded volume was calculated by varying *D*_eff_ from 10 nm to 30 nm, and simulations of the composite NR–CNC systems were performed at *D*_eff_ values of 10, 15, 20, 25, and 30 nm. All simulations were carried out using Origin software 2025b (OriginLab, Northampton, MA, USA).

## 3. Results and Discussions

### 3.1. Self-Assembly Behavior of Composite NR–CNCs

**Figure 4:**
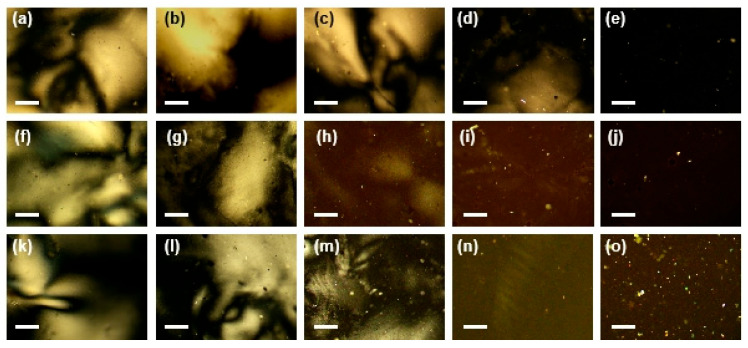
POM images of composite NR–CNC systems at a fixed pH of 11.9 (Ne3), showing the effects of added NR volume (*X*) and aspect ratio. (**a**–**e**) systems with a fixed aspect ratio of 1.3 and varying NR volume: (**a**) *X*_NR1,Ne3_ = 5, (**b**) *X*_NR1,Ne3_ = 10, (**c**) *X*_NR1,Ne3_ = 15, (**d**) *X*_NR1,Ne3_ = 22, and (**e**) *X*_NR1,Ne3_ = 24; (**f**–**j**) Systems with an aspect ratio of 2 and varying NR volume: (**f**) *X*_NR2,Ne3_ = 5, (**g**) *X*_NR2,Ne3_ = 20, (**h**) *X*_NR2,Ne3_ = 26, (**i**) *X*_NR2,Ne3_ = 34, and (**j**) *X*_NR2,Ne3_ = 36; (**k**–**o**) Systems with an aspect of 3.4 and varying NR volume: (**k**) *X*_NR3,Ne3_ = 5, (**l**) *X*_NR3,Ne3_ = 20, (**m**) *X*_NR3,Ne3_ = 26, (**n**) *X*_NR3,Ne3_ = 42, and (**o**) *X*_NR3,Ne3_ = 44. All scale bars represent 200 μm.

Figure 4 illustrates the self-assembly behavior of composite NR–CNCs at a fixed pH of 11.9, influenced by the added volume (*X*) of NRs and their aspect ratios. The systems were observed using POM. In POM imaging, the uniaxial nematic LC phase exhibits a Schlieren texture with bright interference colors, while the isotropic phase appears dark. According to the Michel-Lévy chart, the optical intensity of the nematic phase correlates with interference brightness under polarized light, where duller colors correspond to lower birefringence [32]. As shown in Figure 4a,b, the samples with *X*_NR1,Ne3_ = 5 and *X*_NR1,Ne3_ = 10 display a distinct Schlieren texture, indicating the formation of a uniaxial nematic phase. At *X*_NR1,Ne3_ = 15 (Figure 4c), the Schlieren texture remains visible but appears weaker compared with that at *X*_NR1,Ne3_ = 5. A further increase to *X*_NR1,Ne3_ = 22, results in a sharp decline in the texture contrast (Figure 4d). At *X*_NR1,Ne3_ = 24, the POM image (Figure 4e) appears completely dark, indicating a transition to the isotropic phase. These results demonstrate that the stability of the uniaxial nematic alignment decreases with increasing NR1 volume, and a phase transition from the uniaxial nematic to the isotropic phase occurs at approximately *X*_NR1,Ne3_ = 23. Moreover, as shown in Figure 4f–o, NRs with higher aspect ratio (*AR*_2_ ≈ 2 and *AR*_3_ ≈ 3.4) exhibit similar self-assembly behaviors, though the phase transition occurs at a higher volume of *X*_NR2,Ne3_ = 35 and *X*_NR3,Ne3_ = 43, respectively. These threshold values are significantly larger than those of NR1, indicating that the nematic phase demonstrates enhanced stability against disruption when doped with NRs of higher aspect ratios.

**Figure 5:**
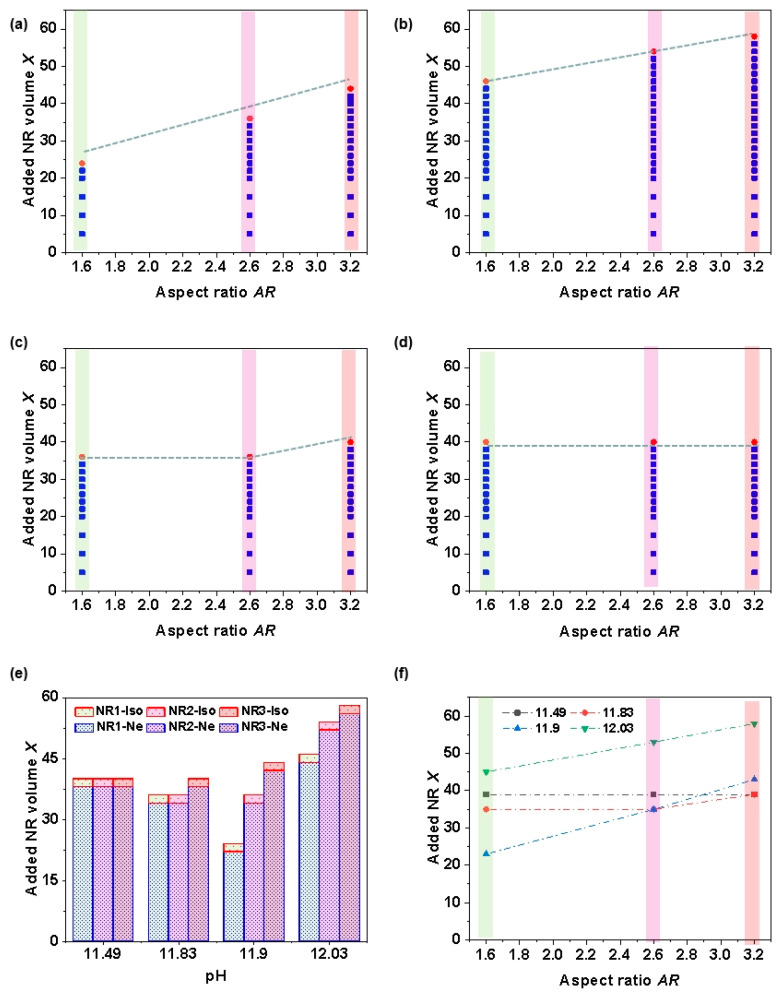
(**a**–**d**) Phase diagram of composite NR–CNC systems at different pH values: (**a**) pH = 11.9, (**b**) pH = 12.03, (**c**) pH = 11.83, and (**d**) pH = 11.49. Red spheres and blue squares represent the isotropic and uniaxial nematic phases, respectively. (**e**) Phase diagram of composite NR–CNC systems as a function of pH and NR aspect ratio. (**f**) Phase transitions between the uniaxial nematic and isotropic phases, highlighting the effects of aspect ratio and pH.

Phase behaviors in Figure 4 are summarized in the phase diagram shown in Figure 5a, where red spheres and blue squares represent isotropic and uniaxial nematic phases, respectively. In addition, POM images of the composite NR–CNC systems at fixed pH values of 12.03, 11.83, and 11.49 are provided in the Supplementary Information (Figures S1–S3), illustrating the effects of added NR volume *X* and the aspect ratio. Figure 5b presents the phase diagram of the composite NR–CNC systems at pH = 12.03, exhibiting a similar trend to that observed at pH = 11.9 (Figure 5a). The transition from the uniaxial nematic to the isotropic phase occurs at a higher NR volume (*X*_NR1,Ne4_ = 45, *X*_NR2,Ne4_ = 53, and *X*_NR3,Ne4_ = 57). In contrast, the phase diagram at pH = 11.83 (Figure 5c) exhibits distinct self-assembly behavior compared to higher pH systems. In this case, the critical transition volumes for NRs with aspect ratios of 1.3 and 2 are identical (*X*_NR1,Ne2_ = *X*_NR2,Ne2_ = 35), which are lower than that of NRs with an aspect ratio of 3.4 (*X*_NR3,Ne2_ = 39). When the pH was further decreased to 11.49, a marked change in the phase behavior was observed (Figure 5d). Under this condition, the transition volumes for all three aspect ratios were identical (*X*_NR1,Ne1_ = *X*_NR2,Ne1_ = *X*_NR3,Ne1_ = 39).

Figure 5e summarizes the phase transition behavior of composite NR–uniaxial nematic CNC systems, and Figure 5f provides a detailed illustration of the transition between uniaxial nematic and isotropic phases. Two dominant trends are evident in Figure 5f: one associated with pH (represented by different lines) and the other with the NR aspect ratio (represented by different bars). At a fixed pH, the NR volume (*X*) required to induce the phase transitions remains nearly constant at lower pH (11.49, black line). In contrast, at higher pH values (12.03 and 11.9, green and blue lines), *X* increases with aspect ratio. For the intermediate pH (11.83, red line), *X* remains constant at smaller aspect ratios (1.3–2) but increases significantly when the aspect ratio increases from 2 to 3. When the aspect ratio is fixed, the pH-dependent trends reveal that for the highest aspect ratio (3.4, third base), *X* increases with pH. For smaller aspect ratios (2 and 1.3, second and first bars), the required *X* at pH ≈ 11.83 is lower than that at pH ≈ 11.49. A similar trend is also observed between pH ≈ 11.9 and pH ≈ 11.83. At pH ≈ 12.03, a larger NR volume is required to induce the phase transition. Therefore, the pH of CNC LCs plays a critical role in the self-assembly behavior of composite NC–CNC systems, while the NR aspect ratio has a more pronounced disruptive effect in the higher pH region.

### 3.2. Composite Effective Excluded-Volume Model

The Onsager excluded-volume theory has been successfully applied to LCs composed of neutral nanorods; however, it shows limitations when applied to composite NR–uniaxial nematic CNC LC systems because both NRs and CNCs possess charged surfaces [33]. To address this issue, a composite effective excluded-volume model is proposed to explain the phase behavior of such composite systems. In this model, the diameter *D* in the Onsager excluded volume-model must be corrected accordingly, and the effective diameter $D_{eff}$ of CNCs can be estimated using the Debye length $d_{e}$.

(4) $$D_{eff}=D(1+d_{e})$$

The effective diameter refers not only to the physical diameter of CNCs but also accounts for the contribution of the electrostatic double-layer repulsion arising from their charged surface. Furthermore, the effective diameter $D_{eff,com}$ of the composite system is defined as

(5) $$D_{eff,com}=D_{eff}+D^{\prime }$$

According to Equation (3), the composite system generally adopts a parallel alignment, and its effective length $L_{eff}$ is determined by the maximum length of either the CNCs L or the doped NRs $L^{\prime }$. Therefore, the effective excluded volume $V_{eff,com}$ can be expressed as

(6) $$V_{eff,com}=\pi {\left(D_{eff}+D^{\prime }\right)}^{2}L_{eff}/4$$

Similarly, the effective aspect ratio ${AR}_{eff,com}$ can be expressed as

(7) $${AR}_{eff,com}=L_{max}/\left(D_{eff}+D^{\prime }\right)$$

Even without the addition of NRs, pure CNC systems undergo a transition to the isotropic phase upon dilution. In an aqueous system consisting solely of CNCs, the effective excluded volume is given by

(8) $$V_{eff,CNCs}=\pi D_{eff}^{2}L/4$$

Based on the parameters in Equations (6) and (8), the excluded volume of the pure CNC system is smaller than that of the composite systems ($V_{eff,CNCs}<V_{eff,com}$). Therefore, due to the influence of the added NRs, the composite systems are more prone to forming isotropic phases. This indicates that, compared with pure CNC systems, the composites exhibit more complex phase behavior, which is crucial for understanding the self-assembly of such composite materials.

**Figure 6:**
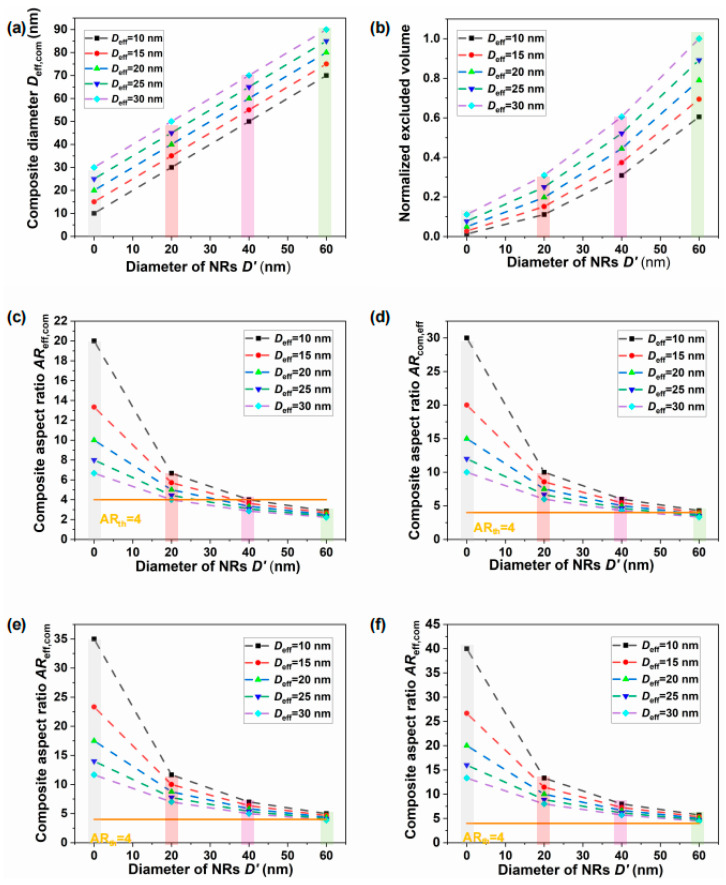
(**a**–**c**) Calculated effective diameter, normalized excluded volume, and aspect ratio of the composite NR–CNC systems with an average CNC length of 200 nm. (**d**–**f**) Calculated aspect ratios of composite NR–CNC systems with NR lengths of 300 nm, 350 nm, and 400 nm. The yellow line indicates the critical aspect ratio (*AR*_th_ = 4), which represents the minimum value required for LC phase formation.

The effective diameters, excluded volumes, and aspect ratios of the composite systems were calculated using Equations (5)–(7), as shown in Figure 6. The first bar represents the simulated values of pure CNC systems (*D*′ = 0 nm), while the second to fourth bars correspond to composite systems containing NRs with diameters of *D*′ = 20, 40, and 60 nm, respectively.

As shown in Figure 6a, the simulated effective diameter *D*_eff,com_ of the composites increases with the effective CNC diameter *D*_eff_, which results from weaker electrostatic interactions at lower pH values (as reflected by the behavior of different lines). The simulated *D*_eff,com_ also increases with the NR diameter *D*′ (as indicated by the variations across different bars). In general, a smaller *D*_eff,com_ corresponds to a larger aspect ratio and higher anisotropy, favoring the formation of a uniaxial nematic phase over the isotropic phase. Moreover, when the NR diameter *D*′ approaches zero, the composite system reduces to a pure CNC system. All effective diameters in the presence of NRs (*D*′ > 0) are larger than those of the pure CNC system, which is consistent with Equations (6) and (8), indicating that the composites are more prone to forming the isotropic phase.

Figure 6b shows the corresponding normalized effective excluded volume. Systems containing NRs with larger aspect ratios exhibit smaller excluded volumes (as seen from differences across bars), which promotes the uniaxial nematic ordering, consistent with the Onsager theory. Similarly, a reduced effective diameter at higher pH values leads to a decrease in excluded volume (as reflected by the behavior of the different lines), further stabilizing the uniaxial nematic order. These simulation results are in good agreement with the pH-dependent behaviors shown in the different lines of Figure 5f, where CNC ordering at higher pH values exhibits greater resistance to NR-induced disruption, while NRs with smaller aspect ratios cause stronger disturbances and facilitate transitions to the isotropic phase.

Figure 6c presents the effective aspect ratio *AR*_eff,com_ of the composites. As shown in Figure 6c, the composite system with *D*′ = 20 nm (*AR*′ ≈ 3.4) has an effective aspect ratio *AR*_eff,com_ > 4, indicating sufficient anisotropy to sustain uniaxial nematic ordering without significant disruption from the doped NRs. This trend is consistent with the aspect ratio behavior illustrated by the third bar in Figure 5f. In contrast, all composites with *D*′ = 60 nm (*AR*′ ≈ 1.3) exhibit effective aspect ratios below 4, indicating strong NR-induced disturbances that hinders LC ordering. For systems with *D*′ = 40 nm (*AR*′ ≈ 2), the effective aspect ratio is partially above 4 (at smaller *D*_eff_ = 10 nm) and partially below 4 (at larger *D*_eff_ = 15–30 nm), suggesting an intermediate level of disruption that becomes more pronounced under weaker electrostatic conditions. These variations in effective aspect ratio may account for the irregular phase transitions observed in the first and second bars of Figure 5f.

In addition, the effective aspect ratio *AR*_eff,com_ of the composites can also be tuned by increasing the NR length until it exceeds that of CNCs. In Equation (7), the parameter *L*_eff_ corresponds to the NR length *L*′ rather than the CNC length *L*. To further examine this effect, simulations were performed to evaluate the effective aspect ratios of composites containing NRs with lengths of *L*′ = 300, 350, and 400 nm, as shown in Figure 6d–f. Figure 6f shows that when *L*′ = 400 nm, all effective aspect ratios exceed 4, indicating that NRs of this length satisfy the Onsager criterion for nematic ordering. However, as illustrated in Figure 6d,e, even with increasing NR length, the aspect ratios remain below the threshold for NRs with larger diameters (e.g., 60 nm, fourth bar). The simulated effective excluded volumes and aspect ratios indicate that the formation of uniaxial nematic phases remains challenging in these systems.

### 3.3. Influence Mechanism of pH and Aspect Ratio on the Self-Assembly of Composite NR–Uniaxial Nematic CNC Systems

**Figure 7:**
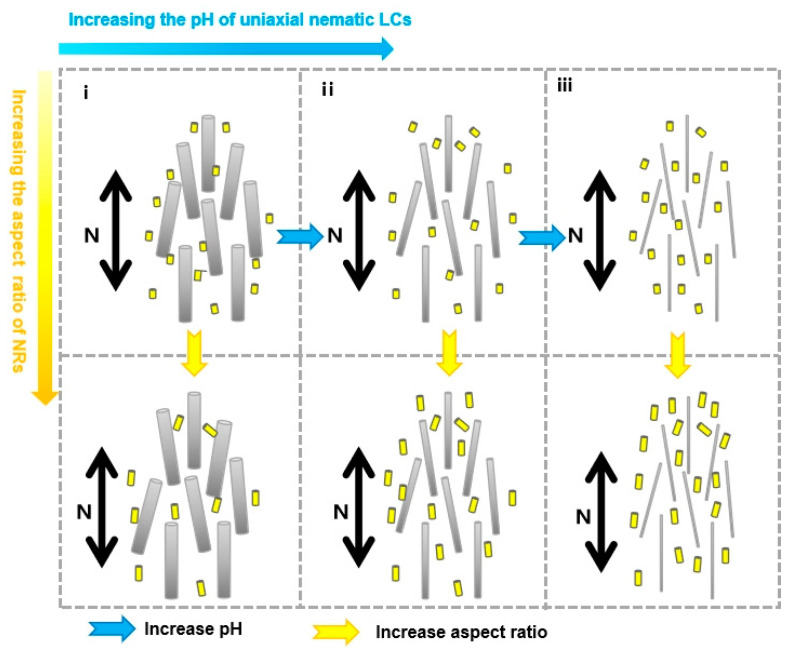
Schematic phase diagrams of composite NR–uniaxial nematic CNC systems with increasing CNC pH and NR aspect ratio: (**i**) low, (**ii**) moderate, and (**iii**) high pH. The black arrow indicates the director **N** of the uniaxial nematic CNC ordering. Yellow and blue thick arrows represent the increasing trends of NR aspect ratio and CNC pH, respectively. Yellow and silver rods denote the added NRs and CNCs, respectively.

Figure 7 illustrates schematic representations of the composite NR–uniaxial nematic CNC systems as a function of increasing CNC pH and NR aspect ratio. Figure 7i–iii show the trend of increasing pH (indicated by blue arrows), while the first and second rows represent the trend of increasing aspect ratio (indicated by yellow arrows).

As illustrated in Figure 7i, at lower pH values, the larger effective diameter reduces the influence of NRs on the excluded volume. The phase diagram of composite NR–CNC systems at pH = 11.46 (Figure 5d) shows that NRs of different aspect ratios exhibit similar phase transition behavior. This observation is consistent with previous findings, which suggest that CNC orientations were essentially unaffected by NRs [23]. With increasing pH in composite NR–CNC systems, enhanced electrostatic interactions decrease the effective diameter. Under these conditions, NRs with larger aspect ratios reduce the excluded volume of the composite system, while NRs with smaller aspect ratios exert only a limited influence on the phase behavior (Figure 7ii). This behavior is confirmed by the phase diagram at pH = 11.83 (Figure 5c), where NRs with larger aspect ratios (2) induce phase transitions at high volumes compared to those with smaller aspect ratios (1.3). At sufficiently high pH values, the effective diameter becomes small enough that even NRs with lower aspect ratios significantly disrupt the uniaxial nematic order. The Phase diagrams at pH = 11.9 and 12.03 (Figure 5a,b) confirm that NRs with larger aspect ratios require higher concentrations to induce phase transitions. This finding is in consistent with the bidisperse Onsager model [34], which predicts that higher aspect ratios favor uniaxial nematic ordering, while lower aspect ratios promote isotropy.

Therefore, the self-assembly behavior of composite NR–uniaxial nematic CNC systems is strongly influenced by both the pH of the CNC LCs and the aspect ratio of the NRs. At higher pH values, the uniaxial nematic alignment of CNCs becomes more resistant to disruption by doped NRs, while NRs with smaller aspect ratios induce stronger perturbations and more readily promote transitions to the isotropic phase. From the perspective of employing composite NR–uniaxial nematic CNC LCs as templates for fabricating nanomaterials and nanostructures, higher-pH conditions generally facilitate the formation of larger uniaxial nematic domains, in which the LC alignment can be more easily preserved without drying-induced defects. Consequently, our findings on high pH composite NR–CNC systems with tunable NR aspect ratios highlight their significant potential for programmable plasmonic nanomaterials and the rational design of advanced functional materials.

## 4. Conclusions

In summary, we have demonstrated the influence of pH and aspect ratio on the self-assembly of composite NR–uniaxial nematic CNC systems. The resulting phase diagram reveals that the uniaxial nematic phase of CNCs becomes more stable at higher pH, while it is more sensitive to disturbance from NRs with smaller aspect ratios. Furthermore, we developed a composite effective excluded-volume model distinct from the conventional Onsager excluded-volume model, in which the interactions between NRs and CNCs are incorporated. In this model, the effective excluded volume is governed by both the effective CNC diameter and the NR aspect ratio. This study elucidates the influence mechanism of pH and aspect ratio on the self-assembly of composite NR–uniaxial nematic CNC systems, in good agreement with experimental observations. This work provides fundamental insights into the use of uniaxial nematic CNC liquid crystals as templates for fabricating advanced nanomaterials and nanostructures, and deepens the understanding of the physical mechanisms governing such composite nanomaterial systems.

## Figures and Tables

**Table 1:** Composition of the 12 composite NR–CNC systems with different pH values and NR aspect ratios.

Composite System	NR1-Ne1	NR2-Ne1	NR3-Ne1	NR1-Ne2	NR2-Ne2	NR3-Ne2	NR1-Ne3	NR2-Ne3	NR3-Ne3	NR1-Ne4	NR2-Ne4	NR3-Ne4
pH	11.49	11.49	11.49	11.83	11.83	11.83	11.9	11.9	11.9	12.03	12.03	12.03
Aspect ratio	1.3	2	3.4	1.3	2	3.4	1.3	2	3.4	1.3	2	3.4

## Data Availability

The original contributions presented in this study are included in the article/Supplementary Materials. Further inquiries can be directed to the corresponding authors.

## Supplementary Materials

The following supporting information can be downloaded at: https://www.mdpi.com/article/10.3390/nano15201594/s1, Figure S1. POM images of composite NR–CNC systems at a fixed pH of 12.03 (Ne4), showing the effects of NR added volume (X) and aspect ratio. Figure S2. POM images of composite NR–CNC systems at a fixed pH of 11.83 (Ne2), showing the effects of NR added volume (X) and aspect ratio. Figure S3. POM images of composite NR–CNC systems at a fixed pH of 11.49 (Ne1), showing the effects of NR added volume (X) and aspect ratio.
