# Supplementary material for: Nematic Alignment of Composite Silver-Coated Gold Nanorods and Cellulose Nanocrystals
Source: Nanomaterials (Basel). 2025 Oct 19;15(20):1594. doi: 10.3390/nano15201594 (PMC12566908; doi:10.3390/nano15201594)
Supplement: Supplementary file 1 [file nanomaterials-15-01594-s001.zip › nanomaterials-3905369-supplementary.pdf]

# Nematic alignment of composite silver coated gold nanorods and cellulose nanocrystals

## Supplementary Materials

**List:**

**Figure S1.** POM images of composite NR−CNC systems at a fixed pH of 12.03 (Ne4), showing the effects of NR added volume (X) and aspect ratio. (a)-(e) systems with a fixed aspect ratio of 1.3 and varying NR volume: (a) $X_{NR1,Ne4}$=5, (b) $X_{NR1,Ne4}$=22, (c) $X_{NR1,Ne4}$=34, (d) $X_{NR1,Ne4}$=44, and (e) $X_{NR1,Ne4}$=46; (f)-(j) Systems with a fixed aspect ratio of 2 and varying NR volume: (f) $X_{NR2,Ne4}$=5, (g) $X_{NR2,Ne4}$=24, (h) $X_{NR2,Ne4}$=36, (i) $X_{NR2,Ne4}$=52, and (j) $X_{NR2,Ne4}$=54 ; (k)-(o) Systems with a fixed aspect of 3.4 and varying NR volume: (k) $X_{NR3,Ne4}$=5, (l) $X_{NR3,Ne4}$=24, (m) $X_{NR3,Ne4}$=38, (n) $X_{NR3,Ne4}$=56, and (o) $X_{NR3,Ne4}$=58.All scale bars represent 200 μm.

**Figure S2.** POM images of composite NR−CNC systems at a fixed pH of 11.83 (Ne2), showing the effects of NR added volume (X) and aspect ratio. (a)-(e) systems with a fixed aspect ratio of 1.3 and varying NR volume: (a) $X_{NR1,Ne2}$=5, (b) $X_{NR1,Ne2}$=10, (c) $X_{NR1,Ne2}$=28, (d) $X_{NR1,Ne2}$=34, and (e) $X_{NR1,Ne2}$=36; (f)-(j) Systems with a fixed aspect ratio of 2 and varying NR volume: (f) $X_{NR2,Ne2}$=5, (g) $X_{NR2,Ne2}$=10, (h) $X_{NR2,Ne2}$=28, (i) $X_{NR2,Ne3}$=34, and (j) $X_{NR2,Ne3}$=36 ; (k)-(o) Systems with a fixed aspect of 3.4 and varying NR volume: (k) $X_{NR3,Ne2}$=5, (l) $X_{NR3,Ne2}$=15, (m) $X_{NR3,Ne2}$=34, (n) $X_{NR3,Ne3}$=38, and (o) $X_{NR3,Ne3}$=40. All scale bars represent 200 μm.

**Figure S3.** POM images of composite NR−CNC systems at a fixed pH of 11.49 (Ne1), showing the effects of NR added volume (X) and aspect ratio. (a)-(e) systems with a fixed aspect ratio of 1.3 and varying NR volume: (a) $X_{NR1,Ne1}$=10, (b) $X_{NR1,Ne1}$=20, (c) $X_{NR1,Ne1}$=28, (d) $X_{NR1,Ne1}$=38, and (e) $X_{NR1,Ne1}$=40; (f)-(j) Systems with a fixed aspect ratio of 2 and varying NR volume: (f) $X_{NR2,Ne1}$=10, (g) $X_{NR2,Ne1}$=20, (h) $X_{NR2,Ne1}$=26, (i) $X_{NR2,Ne1}$=38, and (j) $X_{NR2,Ne1}$=40 ; (k)-(o) Systems with a fixed aspect of 3.4 and varying NR volume: (k) $X_{NR3,Ne1}$=10, (l) $X_{NR3,Ne1}$=15, (m) $X_{NR3,Ne1}$=22, (n) $X_{NR3,Ne1}$=38, and (o) $X_{NR3,Ne1}$=40. All scale bars represent 200 μm.

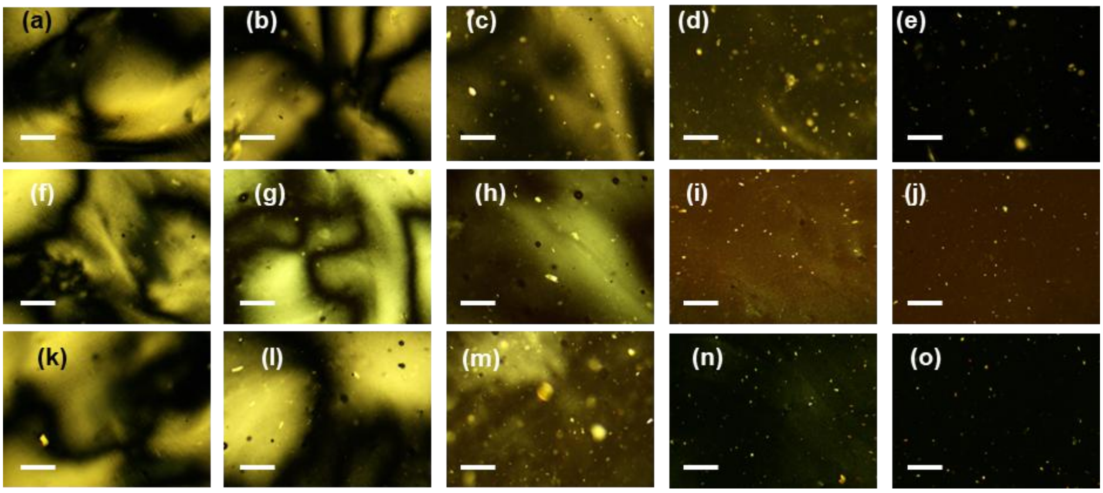


**Figure S1.** POM images of composite NR−CNC systems at a fixed pH of 12.03 (Ne4), showing the effects of NR added volume (X) and aspect ratio. (a)-(e) systems with a fixed aspect ratio of 1.3 and varying NR volume: (a) $X_{NR1,Ne4}$=5, (b) $X_{NR1,Ne4}$=22, (c) $X_{NR1,Ne4}$=34, (d) $X_{NR1,Ne4}$=44, and (e) $X_{NR1,Ne4}$=46; (f)-(j) Systems with a fixed aspect ratio of 2 and varying NR volume: (f) $X_{NR2,Ne4}$=5, (g) $X_{NR2,Ne4}$=24, (h) $X_{NR2,Ne4}$=36, (i) $X_{NR2,Ne4}$=52, and (j) $X_{NR2,Ne4}$=54 ; (k)-(o) Systems with a fixed aspect of 3.4 and varying NR volume: (k) $X_{NR3,Ne4}$=5, (l) $X_{NR3,Ne4}$=24, (m) $X_{NR3,Ne4}$=38, (n) $X_{NR3,Ne4}$=56, and (o) $X_{NR3,Ne4}$=58.All scale bars represent 200 μm.

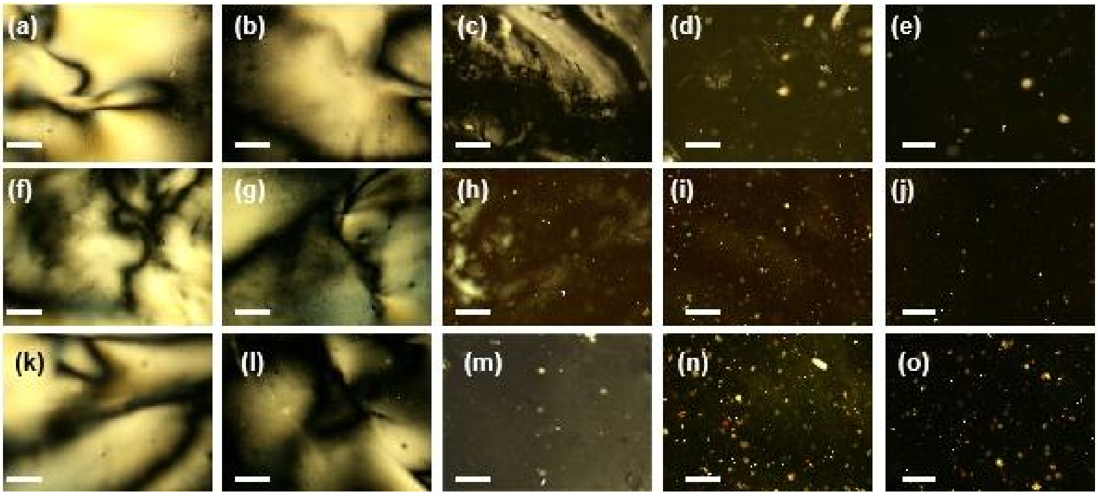


**Figure S2.** POM images of composite NR−CNC systems at a fixed pH of 11.83 (Ne2), showing the effects of NR added volume (X) and aspect ratio. (a)-(e) systems with a fixed aspect ratio of 1.3 and varying NR volume: (a) $X_{NR1,Ne2}$=5, (b) $X_{NR1,Ne2}$=10, (c) $X_{NR1,Ne2}$=28, (d) $X_{NR1,Ne2}$=34, and (e) $X_{NR1,Ne2}$=36; (f)-(j) Systems with a fixed aspect ratio of 2 and varying NR volume: (f) $X_{NR2,Ne2}$=5, (g) $X_{NR2,Ne2}$=10, (h) $X_{NR2,Ne2}$=28, (i) $X_{NR2,Ne3}$=34, and (j) $X_{NR2,Ne3}$=36 ; (k)-(o) Systems with a fixed aspect of 3.4 and varying NR volume: (k) $X_{NR3,Ne2}$=5, (l) $X_{NR3,Ne2}$=15, (m) $X_{NR3,Ne2}$=34, (n) $X_{NR3,Ne3}$=38, and (o) $X_{NR3,Ne3}$=40. All scale bars represent 200 μm.

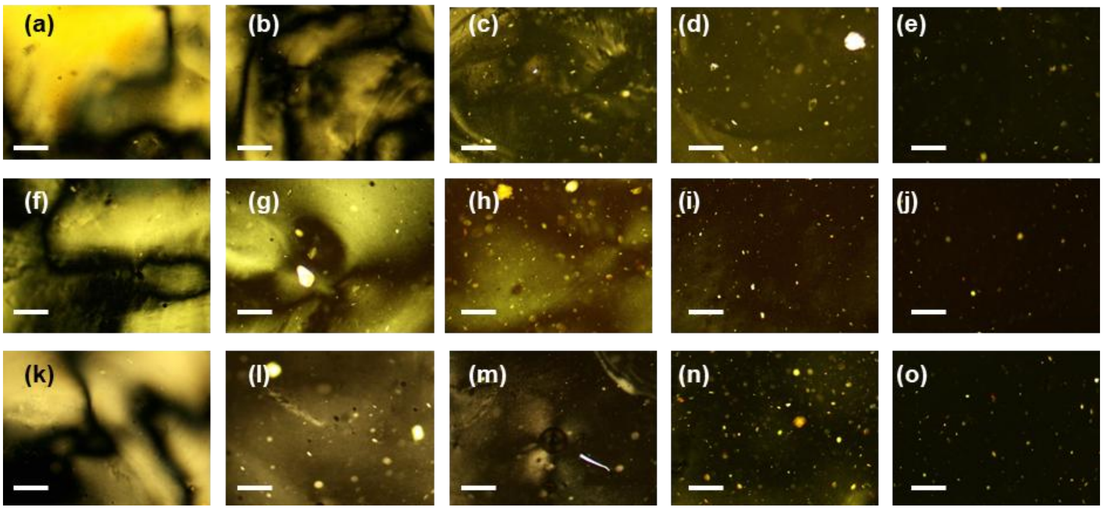


**Figure S3.** POM images of composite NR−CNC systems at a fixed pH of 11.49 (Ne1), showing the effects of NR added volume (X) and aspect ratio. (a)-(e) systems with a fixed aspect ratio of 1.3 and varying NR volume: (a) $X_{NR1,Ne1}$=10, (b) $X_{NR1,Ne1}$=20, (c) $X_{NR1,Ne1}$=28, (d) $X_{NR1,Ne1}$=38, and (e) $X_{NR1,Ne1}$=40; (f)-(j) Systems with a fixed aspect ratio of 2 and varying NR volume: (f) $X_{NR2,Ne1}$=10, (g) $X_{NR2,Ne1}$=20, (h) $X_{NR2,Ne1}$=26, (i) $X_{NR2,Ne1}$=38, and (j) $X_{NR2,Ne1}$=40 ; (k)-(o) Systems with a fixed aspect of 3.4 and varying NR volume: (k) $X_{NR3,Ne1}$=10, (l) $X_{NR3,Ne1}$=15, (m) $X_{NR3,Ne1}$=22, (n) $X_{NR3,Ne1}$=38, and (o) $X_{NR3,Ne1}$=40. All scale bars represent 200 μm.
